# T cell immunity following COVID-19 vaccination in adult patients with primary antibody deficiency – a 22-month follow-up

**DOI:** 10.3389/fimmu.2023.1146500

**Published:** 2023-05-09

**Authors:** Antti Hurme, Pinja Jalkanen, Minna Marttila-Vaara, Jemna Heroum, Heidi Jokinen, Saimi Vara, Oona Liedes, Johanna Lempainen, Merit Melin, Ilkka Julkunen, Leena Kainulainen

**Affiliations:** ^1^ Department of Infectious Diseases, Turku University Hospital and University of Turku, Turku, Finland; ^2^ Institute of Biomedicine, University of Turku, Turku, Finland; ^3^ Department of Internal Medicine, Lapland Central Hospital, Rovaniemi, Finland; ^4^ Clinical Microbiology, Turku University Hospital, Turku, Finland; ^5^ Department of Health Security, Finnish Institute for Health and Welfare, Helsinki, Finland; ^6^ Department of Paediatrics and Adolescent Medicine, Turku University Hospital and University of Turku, Turku, Finland

**Keywords:** common variable immunodeficiency, COVID-19, vaccine, T cell mediated immunity, humoral immunity

## Abstract

Primary antibody deficiencies, such as common variable immunodeficiency (CVID), are heterogenous disease entities consisting of primary hypogammaglobulinemia and impaired antibody responses to vaccination and natural infection. CVID is the most common primary immunodeficiency in adults, presenting with recurrent bacterial infections, enteropathy, autoimmune disorders, interstitial lung diseases and increased risk of malignancies. Patients with CVID are recommended to be vaccinated against SARS-CoV-2, but there are relatively few studies investigating humoral and cellular responses to immunization. We studied the dynamics of humoral and cell-mediated immunity responses up to 22 months in 28 patients with primary immunodeficiency and three patients with secondary immunodeficiency receiving ChAdOx1, BNT162b2 and mRNA-1273 COVID-19 vaccines. Despite inadequate humoral response to immunization, we demonstrate a robust T cell activation likely protecting from severe COVID-19.

## Introduction

Patients with primary immunodeficiency (PID) and secondary immunodeficiencies are at an increased risk of getting a severe acute coronavirus 2 (SARS-CoV-2) infection and a long-lasting COVID-19 (1, 2). In healthy immunocompetent adults, COVID-19 vaccinations induce humoral and cell-mediated responses (3, 4), of which neutralizing antibodies are associated with protective immunity (5). In addition, both CD4+ and CD8+ T cells are needed to generate a robust humoral response (6–9) and antigen-specific T cells are shown to limit the disease severity (10). Previous studies on immunocompromised patients have demonstrated weak or absent antibody responses but robust cell-mediated responses after two doses of the COVID-19 vaccine (11, 12). However, further studies are required on the longevity of the cell-mediated responses in immunocompromised patients.

The most common group of PIDs is common variable immunodeficiency (CVID), characterized by a functional defect of B cells to maturate to plasma cells and decreased age-adjusted serum IgG, IgA, or IgM antibody levels. CVID patients are susceptible to recurrent infections, autoimmune manifestations, and malignancies, and they are known to mount suboptimal responses against vaccines (13). In addition, patients may have defects in T cell functions, leading to a more severe late-onset common immunodeficiency (14). Another group of primary antibody deficiencies is X-linked agammaglobulinemia (XLA) caused by Bruton kinase (Btk) mutations leading to an arrest of B cell maturation in the bone marrow (15). In a small cohort study, XLA patients had a better outcome after SARS-CoV-2 infection than CVID patients (16).

Secondary hypogammaglobulinemia (SHG) is an immune deficiency caused by an external factor. The causative agents can be infections, such as HIV, malnutrition, chronic kidney disease, severe liver disease or severe loss of protein in conditions such as nephrotic syndrome or protein-losing enteropathy (17, 18). Chemotherapeutic agents, glucocorticoid treatment, and radiotherapy can also cause transient defects in immune functions. However, persistent SHGs are mainly caused by biological therapies targeting B cells, such as rituximab, an anti-CD20 monoclonal antibody (19, 20), or directly by chronic or acute lymphoproliferative or myeloproliferative diseases (21, 22).

In the present study, we have conducted a 22-month follow-up of humoral and cell-mediated immune responses in one to four times COVID-19-vaccinated primary and secondary immunodeficiency patients. We show that while only 24% of the patients have detectable anti-S1 antibodies after the second vaccine dose, 94% generate a robust CD4+ T cell response against spike protein of wild-type strain and Delta and Omicron BA.2 variants. In addition, we demonstrate that spike-specific T cell responses are maintained at least three months after the third vaccination.

## Materials and methods

### Study participants

This is a prospective follow-up study. In January 2020, PID and SHG patients treated and followed up at Turku University Hospital, Turku, were invited to participate in the study. The diagnosis of CVID and XLA was based on international diagnostic criteria (23). In addition, three SHG patients were included. Almost all patients (30/31) were on regular immunoglobulin replacement therapy; 9 IVIG and 21 SCIG. Patient age, medications, serum immunoglobulin concentrations, and T cell counts at disease onset have been described in **Table 1**.

**Table 1:** Patient characteristics.

Patient code	sex	age at DG	age	DG	IGRT	IgG at dg (g/l)	IgA at dg (g/l)	IgM at dg	through IgG	CD4 count (E9/l)	CD8 count (E9/l)	CD4/CD8 ratio	Medication	Comorbidities
1	f	24	31	CVID	scig	3,9	0,1	2,2	9,1	0,76	0,26	2,92	no	BE
2	f	31	34	CVID	scig	3,2	0,3	0,5	9,7	0,96	0,47	2,04	no	no
3	f	23	36	CVID	ivig	4,1	0,2	0,3	7,7	0,09	0,01	6,25	SMZ-TMP, carvedilol, spironolactone, insulin	T1DM, chronic liver disease, ITP, chronic norovirus infection
4	f	5	36	CVID	scig	1,7	0,1	0,1	7,6	0,63	0,70	0,9	no	BE
5	m	29	37	CVID	scig	4,8	0,9	0,3	7,3	0,45	0,14	3,2	levetirazetam	epilepsy
6	f	30	44	CVID	ivig	0,7	<0,1	<0,2	7,8	0,60	0,30	2,2	no	BE
7	f	40	45	CVID	scig	0,3	0,9	0,3	12	1,13	0,74	1,52	no	no
8	f	26	52	CVID	scig	<0,5	<0,1	<0,2	9,3	0,39	0,48	0,7	bisoprolol, lakosamide	SSS, epilepsy, BE
9	m	32	53	CVID	ivig	3,4	<0,2	<0,2	9	0,13	0,07	2,8	SMZ-TMP, prednisolon	GLILD, ITP, died of SARS-CoV-2
10	f	45	53	CVID	ivig	2,8	<0,1	<0,2	10,8	0,65	0,32	2,03	acetazolamide, ciclesonide	Chronic uveitis, BE, asthma
11	f	40	53	CVID	scig	<0,1	<0,5	1,0	8,4	0,27	0,25	1,09	insulin, losartan	T1DM, HTN
12	m	41	54	CVID	scig	<0,5	0,5	<0,2	7,5	0,41	0,34	1,2	fluticasone furoate	asthma, AF, vitiligo
13	m	24	55	CVID	scig	3,5	0,1	0,4	8,9	1,93	4,16	0,46	ramipril	HTN
14	m	28	59	CVID	scig	1.0	<0,1	<0,2	8,9	0,31	0,22	1,4	fluticasone furoate, thyroxin	asthma, hypothyroidism, colorectal cancer
15	f	59	61	CVID	scig	<0,1	1,3	<0,2	5,7	0,93	0,49	1,9	methotrexate, mesalazine, prednisolon	CU, RA
16	m	18	61	CVID	ivig	2,2	<0,2	<0,2	10,6	0,70	2,23	0,3	no	no
17	f	33	65	CVID	scig	0,3	<0,1	<0,1	8,8	0,33	0,64	0,51	valsartan, hydrochlorthiatzide	HTN, BE, sarcoidosis, OSA
18	m	49	67	CVID	scig	<0,5	<0,1	<0,2	9,4	0,83	0,62	1,3	no	BE, Bowen’s disease
19	f	55	70	CVID	scig	2,7	<0,1	<0,2	10,4	0,53	0,44	1,4	rivaroxaban	AF, lymphocytic colitis, PA
20	f	68	71	CVID	ivig	<0,5	<0,2	<0,2	9,1	1,50	1,49	1	leflunomide, alendronat	Reactive arthritis, breast cancer
21	m	67	71	CVID	scig	<0,1	<0,5	<0,2	6,8	0,26	0,25	1,04	SMZ-TMP, prednisolon	Possible sarcoidosis, osteoporosis
22	f	61	74	CVID	ivig	1,8	<0,2	<0,2	8,7	0,55	0,19	1,7	no	BE, died of CMV
23	f	72	77	CVID	scig	<0,1	<0,5	<0,2	7,6	1,66	0,56	2,95	insulin, prednisolon	sarcoidosis, T2DM, HTN
24	m	1	58	XLA	scig	1,0	<0,1	0,1	7,2	1,07	0,63	1,68	no	BE
25	f	23	26	SAD	scig	<0,1	8,7	0,7	9,4	1,63	1,06	1,54	no	endometriosis
26	m	62	67	SAD	scig	1,5	9,8	0,8	13,1	0,62	1,44	0,43	no	no
27	f	50	57	SHG	scig	<0,1	1,3	<0,2	6,3	0,23	0,78	0,3	R-CHOP (2014)	B cell lymphoma
28	f	50	57	SHG	scig	0,2	3,4	0,4	7,9	0,08	0,08	0,96	cytarabine, methotrexate, rituximab, SCT	B cell lymphoma
29	m	66	71	SHG	ivig	0,2	3,7	<0,2	6,9	0,21	0,15	1,44	SMZ-TMP, voriconazole	Chronic lymphocytic leukemia
30	f	59	77	ScD	ivig	5,4	7,9	3,2	11,3	0,75	0,78	0,96	cephalexin	Cecum cancer
31	f	72	75	SAD	ab prof	7,1	1,7	1,8	7,3	0,93	0,49	1,91	prednisolone, azithromycin	ABPA, bronchiectasies

Normal values:

CD4 count 0,7-1,1 E9/l.

CD8 count 0,5-0,9 E9/l.

The control group consisted of healthcare workers (HCWs, n=10) vaccinated two times with the BNT162b2 COVID-19 mRNA vaccine with a 3-week dose interval. HCW samples have been analyzed previously using an identical laboratory protocol (24).

### Ethics

The study protocol was approved by the Ethics Committee of the Hospital District of Southwest Finland (PID and SHG patients; permission number ETMK 89/1800/2021 and EudraCT 2021-004891-33, HCWs; ETMK 19/1801/2020 and EudraCT 2021-004419-14). All participants provided a written informed consent. The study adheres to the principles of the declaration of Helsinki.

### Follow-up of the PID and SHG patients

COVID-19 vaccinations started in December 2020 in Finland, and during the follow-up, three different vaccines were used for vaccination: mRNA vaccines BNT162b2 (Pfizer-BioNTech) and mRNA-1273 (Moderna), and adenovirus vector vaccine ChAdOx1-S (AstraZeneca). The combination of vaccines used in different patients is detailed in **Supplementary Table 1**.

Study participants were informed of the schedule when the blood samples were to be drawn, and they informed the investigators when they received the vaccinations and which vaccine was given. In addition, the participants were asked to report possible side effects after receiving vaccinations. The investigators could not influence which vaccines the participants received. The blood samples were drawn up to two weeks before and three weeks and three months after the first, second, third and fourth vaccinations.

### Cell isolation

For each sample, we collected 10 ml blood for serum separation and 20 ml heparinized blood for lymphocyte separation. Peripheral blood mononuclear cells (PBMCs) were isolated using Ficoll-Paque PLUS (GE Healthcare) density gradient centrifugation according to the manufacturer’s protocol. Cells were counted with TC20 automated cell counter (BioRad), and cell viability was assessed with trypan blue dye (BioRad). Isolated PBMCs were suspended into a concentration of 10 x10^6^ cells/mL in a freezing medium containing 10% DMSO and 10% human AB serum (Sigma-Aldrich) and gradually frozen to -135°C until further use.

### Cell culture and stimulations

Cryopreserved PBMCs were rapidly thawed and washed with culture media (RPMI-1640 culture medium (Gibco) supplemented with 10% human AB serum (Sigma), 2mM L-glutamine, and penicillin/streptomycin) and rested overnight at a concentration of 5x10^6^ cells/ml as previously described (4). After resting, the cells were plated into 96-well U-bottom plate (Thermo) and stimulated with 0.5µg/ml of peptide pools covering the whole SARS-CoV-2 spike protein of wild-type (wt) strain (PM-WCPV-S-1), Delta variant (PM-SARS2-SMUT06-1), and Omicron BA.2 (PM-SARS2-SMUT09-1) variant dissolved in DMSO. Peptide pools consisted of 15mers with 11mer overlaps. Tetanus toxoid (AJ Vaccines) was used as a positive control, and DMSO in culture media was used as a negative control. Cells were incubated for 48h at +37°C and 5% CO2.

### Flow cytometry

Stimulated PBMCs were washed and stained according to the protocol described in detail previously (4). Cells were stained with Zombie green dye (BioLegend) and a collection of antibodies against lymphocyte surface markers CD45, CD3, CD4, CD8, CD69, CD134, CD137, CD45RA, and CCR7 (**Supplementary Table 2**). T cell subtypes were characterized with NovoCyte Quanteon Flow Cytometer (Agilent Technologies Inc) and analyzed with NovoExpress v1.5.0 (Agilent Technologies Inc).

T cell activation was shown as stimulation index (SI) values that were calculated by dividing the percentage of cells positive for activation induced markers (AIM+) after spike peptide stimulation with the percentage of AIM+ cells after stimulation with DMSO. If there were no activated cells after stimulation with DMSO, the stimulation index was marked as the lowest value of the participant, and SI values <0.5 were marked as 0.5 SI. Samples with a CD3+ T cell count <8000 cells in sample volume, or an abnormal or missing response to tetanus toxoid in a single sample (average SI +/- 2SD or SI<1) were excluded from the analysis. A positive result (an increased stimulation index) was determined as previously described (4).

### Cytokine detection using Luminex

Supernatants of the stimulated PBMCs in 96‐well plates were measured for the levels of secreted cytokines (IFN-γ, IL-2, IL-4 and TNF-α) and effector molecules (granzyme B and perforin) using the MILLIPLEX^®^ MAP Kit HCD8MAG‐15K (Millipore^®^), according to manufacturer’s instructions with Luminex MAGPIX magnetic bead analyzer (Luminex^®^ Corporation). A median fluorescent intensity value was counted for seven diluted standards to calculate the concentration using a 5-parameter logistic regression. Only samples in the linear range were quantified. For statistical analyses, samples below the lowest standard in the linear range were given half of the value of the lowest standard (5 pg/ml for IFN-γ, 7 pg/ml for IL-2, 10 pg/ml for IL-4, 0.5 pg/ml for TNF-α, 1 pg/ml for granzyme B and 50 pg/ml for perforin). Samples over the highest standard in the linear range were given the highest value of the standard (5000 pg/ml for IFN-γ, 7500 pg/ml for IL-2, 10 000 pg/ml for IL-4, 2000 pg/ml for TNF-α, 5000 pg/ml for granzyme B and 50 000 pg/ml for perforin). Standards with a standard deviation of less than 20% for the duplicates were accepted. According to the kit manufacturer, if there were less than 35 beads in the well, the samples could not be given a reliable concentration, and thus, those samples were discarded from the final analysis.

### SARS-CoV-2 S1 and N protein antibody enzyme immunoassay

An in-house EIA was used to analyze the levels of SARS-CoV-2 S1- and N-specific IgG antibodies as described previously (25). Briefly, 96-well plates were coated with purified recombinant SARS-CoV-2 antigens (3.5 µg/ml of S1 and 2.0 µg/ml of N) and blocked with assay buffer (5% swine serum and 0.5% Tween-20 in PBS). Serum samples were diluted to 1:300 in assay buffer, and the amount of bound IgG was quantified with an absorbance measurement at a 450nm wavelength. Linear interpolation between the optical density (OD) values of the known positive (100 EIA units) and negative control (0 EIA units) serum samples was used to convert the OD values to EIA units. The cut-off values for seropositivity were as described previously (26).

### Statistical analysis

Data were analyzed with GraphPad Prism (version 8). Non-paired samples were tested with Mann-Whitney U-test, and paired samples were tested with Wilcoxon signed rank test or the Friedman test, followed by Dunn’s multiple comparison test. The Kruskal-Wallis test was used when participants lacked samples from individual time points. All tests were two-sided, and p-values <0.05 were considered statistically significant. Correlations were analyzed with Spearman’s correlation test. Statistical analyses used in the individual comparisons are detailed further in the figure legends.

## Results

### Patient characterization

The patient cohort consisted of 31 COVID-19 vaccinated hypogammaglobulinemia patients (20 female, 11 male): 23 common variable immunodeficiency patients (CVID), one X-linked agammaglobulinemia (XLA), three specific antibody deficiency (SAD), one IgG subclass deficiency (IgG ScD) patient, and three patients with secondary hypogammaglobulinemia (SHG) caused by treatment with rituximab, an anti-CD20 monoclonal antibody. In two cases, rituximab was used for B-cell lymphoma and in one case for chronic lymphocytic leukemia (CLL). The median age at diagnosis was 33 years (range 1-72 years), and the median age during this study was 55years (range 26-77 years). The average through IgG level was 8.8 g/L (5.7-13.1 g/L).

Genetic cause was identified in only three CVID patients: CF1 c.982G>A HET Gly382Arg in patient 3 and PIK3D Het c.2389A>G(met797Val) in patient 8 with CVID and BTK-mutation in patient 24 with XLA. Bronchiectasis was the most common comorbidity in CVID patients (9/23), and seven patients had autoimmune manifestations. Nine patients had SARS-CoV-2 infection during the study period, and one died of COVID-19.

**Figure 1:**
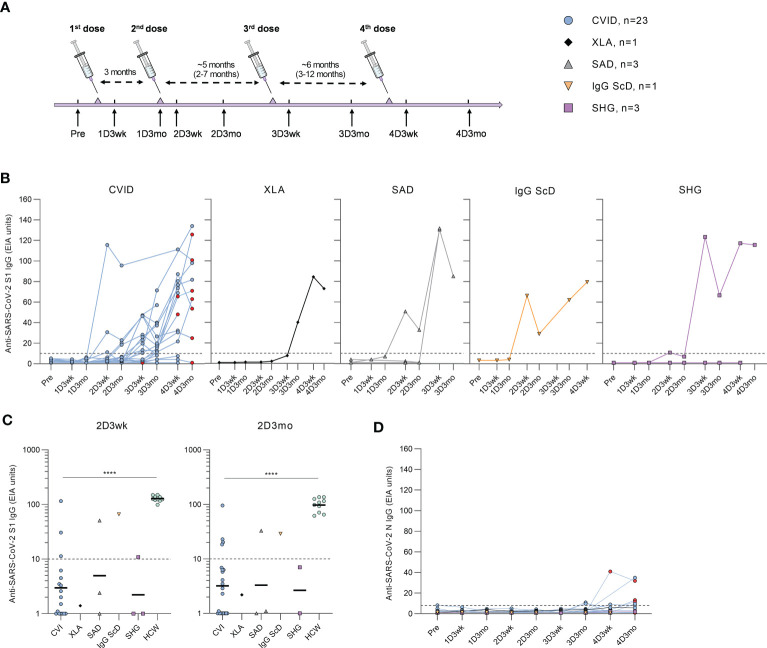
Humoral responses in patients with hypogammaglobulinemia. **(A)** Vaccination and sample collection schedule. Average dose intervals are defined above the timeline and range is in parenthesis. **(B)** Anti-SARS-CoV-2 S1-specific antibody responses by different types of hypogammaglobulinemia (CVID, XLA, SAD, IgG ScD, and SHG) in samples collected before vaccination (Pre) and three weeks and three months after each vaccination. Red dots represent samples collected after SARS-CoV-2 infection. **(C)** Comparison of anti-S1 IgG levels in samples collected three weeks (2D3wk) and three months (2D3mo) after the second dose from hypogammaglobulinemia patients and healthy controls (HCWs). The antibody levels in CVID and HCW group were compared with Mann-Whitney U-test. Values for HCW controls were obtained from our previous study with an identical laboratory analysis protocol (4). **(D)** Anti-SARS-CoV-2 N-specific antibody responses by different types of hypogammaglobulinemia (CVID, XLA, SAD, IgG ScD, and SHG). Dotted lines represent the cut-off values. ****p<0.0001.

Patients received one to four doses of COVID-19 vaccines (**Supplementary Table 1**), and PBMC and serum samples were collected a maximum of two weeks before the first vaccination and three weeks and three months after the first, second, third, and fourth vaccinations (**Figure 1A**).

### Humoral responses in patients with hypogammaglobulinemia

Antibodies against SARS-CoV-2 S1 and N proteins were analyzed from serum samples collected from hypogammaglobulinemia patients (n=31) and a group of immunocompetent health care workers (HCWs, n=10). After the first vaccine dose, none of the hypogammaglobulinemia patients had anti-S1 antibodies. However, three weeks after the second vaccine dose, anti-S1 antibodies were detected in 6/25 (24%) patients (three CVID, one SAD, one IgG ScD, and one SHG), and three months after the second dose, the antibody response was detected in 6/29 (21%) patients (four CVID, one SAD, and one IgG ScD) (**Figure 1B**). Antibody levels in CVID patients were significantly lower than in HCWs (p<0.0001, **Figure 1C**).

After the third and fourth vaccine doses, the levels of anti-S1 antibodies were detectable in all disease groups (**Figure 1B**). Three weeks and three months after the third dose, S1-specific antibodies were detected in 15/21 (71%) (11 CVID, one XLA, one SAD, one SHG) and 14/21 (67%) (10 CVID, one XLA, two SAD, one IgG ScD and one SHG) of the patients, respectively. Three weeks and three months after the fourth dose, 14/19 (74%) (11 CVID, one XLA, one IgG ScD, and one SHG) and 11/12 (92%) (nine CVID, one XLA, and one SHG) of the patients had measurable anti-S1 IgG antibodies, respectively. Interestingly, most CVID patients receiving subcutaneous immunoglobulin replacement therapy (81%) had detectable levels of anti-S1 antibodies, while only three out of seven (43%) patients receiving intravenous immunoglobulin replacement therapy were anti-S1 IgG positive (**Supplementary Figure 1**).

Nine CVID patients had SARS-CoV-2 infection during the study period. All infections were during 2022 when Omicron variant was the main variant in Finland. Four patients had an increase in anti-S1 antibody levels after infection, while three had decreasing anti-S1 antibody levels, and two remained seronegative. Three of the patients with anti-S1 antibodies had an increase in anti-SARS-CoV-2 nucleoprotein (N) antibodies after the infection (**Figure 1D**). However, four CVID patients with no known SARS-CoV-2 infection were also seropositive for anti-N antibodies.

### Activation of T cells in patients with hypogammaglobulinemia

**Figure 2:**
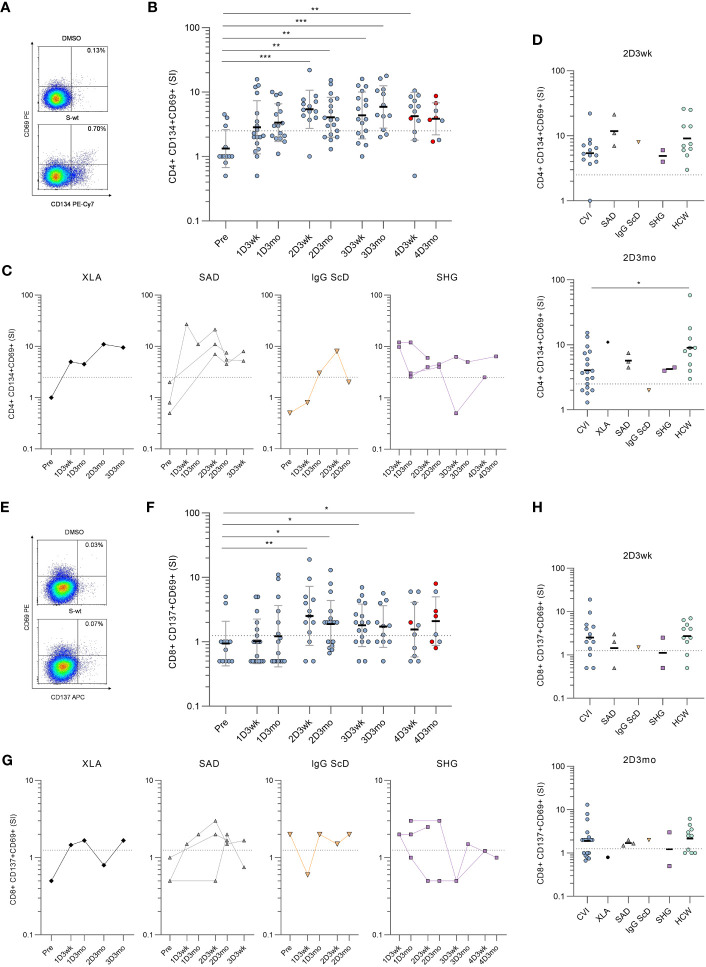
Spike-specific CD4+ and CD8+ T cell responses in patients with hypogammaglobulinemia. **(A)** Representative flow cytometry plots and gating of DMSO and SARS-CoV-2 wild-type (S-wt) spike -specific CD4+ T cells determined by the dual expression of CD69 and CD134. **(B)** S-wt-specific CD4+ T cell responses in CVID patients. Red dots represent samples collected after SARS-CoV-2 infection. **(C)** S-wt-specific CD4+ responses in other hypogammaglobulinemia groups (XLA, SAD, IgG ScD, and SHG). **(D)** Comparison of S-wt-specific CD4+ responses in 2D3wk and 2D3mo samples between patients with hypogammaglobulinemia and healthy controls (HCWs). Values for healthy controls were from our previous study (4). Statistical significance between CVID patients and healthy HCW controls was determined with Mann-Whitney U-test. **(E)** Representative flow cytometry plots of DMSO and S-wt-specific CD8+ T cells determined by the dual expression of CD69 and CD137. **(F)** S-wt-specific CD8+ T cell responses in CVID patients. Kruskall-Wallis test followed by Dunn’s multiple comparisons test was used to compare pre-vaccination samples with samples collected after vaccinations. **(G)** S-wt-specific CD8+ responses in other hypogammaglobulinemia groups (XLA, SAD, IgG ScD, and SHG). **(H)** Comparison of CD8+ responses between 2D3wk and 2D3mo samples in patients with hypogammaglobulinemia and healthy controls (HCWs). The dotted lines represent the cut-off values. *p<0.05; **p<0.01; ***p<0.001.

Next, SARS-CoV-2 spike (S)-specific CD4+ and CD8+ T cell responses were assessed from hypogammaglobulinemia patients (n=31) and immunocompetent HCWs (n=10) with activation induced marker (AIM) assay. In the AIM assay, PBMCs were stimulated with peptide pools covering the entire S protein of wild-type (wt) strain, Delta variant, or Omicron BA.2 variant. S-specific CD4+ T cell responses were determined by the dual expression of CD69 and CD134, and CD8+ T cell responses by the dual expression of CD69 and CD137 activation markers (**Supplementary Figure 2** and **Figures 2A, E**).

Unlike in humoral responses, SARS-CoV-2 wt S-specific CD4+ T cell responses were detected in 12/23 (52%) patients three weeks and 15/23 (65%) patients three months after the first vaccine dose (**Figure 2B, C**). After the second vaccine dose (three-week-timepoint) S-specific CD4+ T cell responses were detected in all except one CVID patient (18/19, 95%), and the response in CVID patients was comparable to the response seen in HCWs (**Figure 2D**). However, after three months, the responses declined >2.5 SI in three CVID patients, three SAD patients, and one IgG ScD, and at the three-month-timepoint, the response was detected in 18/25 (72%) patients. Furthermore, the responses in CVID patients had declined more than in HCWs (p<0.05; **Figure 2D**).

Three weeks and three months after the third vaccine dose, the S-specific CD4+ T cell response was detected in 14/21 (67%) and 12/14 (86%) patients, respectively. The response was seen in 16/20 CVID patients, 1/2 SHG patient, and all XLA and SAD patients in one or both time points (**Figures 2B, C**). After the fourth vaccine dose, a positive response was detected in 9/13 (69%) patients three weeks after and in 7/9 (78%) patients three months after the fourth vaccination. In CVID patients, no statistically significant difference was detected between responses after the second and fourth vaccination. However, variation is seen between individual patients’ responses, and SARS-CoV-2 infection does not have a clear effect on S-specific CD4+ T cell response. Different COVID-19 vaccine combinations did not generate different S-specific CD4+ or CD8+ T cell responses (**Supplementary Figure 3**).

Notably, six CVID patients and all SHG patients had CD4+ T cell counts <350 cells/mm^3^ at the time of the diagnosis, while all XLA, IgG ScD, and SAD patients had CD4+ T cell counts >350/mm^3^. With CVID patients, a low CD4+ T cell count led to a more inadequate CD4+ response against SARS-CoV-2 wt spike peptide pool and tetanus toxoid in most of the time points, but not in three weeks after the third or fourth vaccine dose (**Supplementary Figure 4**).

CD8+ T cell activation was lower than CD4+ T cell activation when stimulated with SARS-CoV-2 wild-type spike peptide pool.S-specific CD8+ T cell responses were detected in 9/23 (39%) three weeks and in 11/22 (50%) patients three months after the first vaccine dose (**Figure 2F**). The responses increased after the second dose, and 14/19 (74%) and 16/25 (64%) patients had a positive S-specific CD8+ T cell response three weeks and three months after the second dose, respectively. The response was seen in both SAD patients and in patient with IgG ScD. For SHG patients, the activation was observed in 1/2 patients and there was no CD8+ activation for XLA patient (**Figure 2G**). There was no statistical difference between T cell responses in CVID patients and HCWs three weeks and three months after the second dose (**Figure 2H**). Three weeks and three months after the third vaccination, S-specific CD8+ T cell responses were detected in 12/21 (57%) and in 9/13 (69%) patients, respectively. After the fourth vaccination, CD8+ responses were found only in 5/11 (45%) and 5/9 (56%) of the patients at three weeks and three months after vaccination, respectively.

To further assess the long-term memory T cell function, we analyzed memory T cell populations for four patients after the fourth vaccination. We measured the expression of CD45RA and CCR7 to differentiate between central (T_CM_; CD45RA-CCR7+), effector (T_EM_, CD45RA-CCR7-) and terminally differentiated effector (T_EMRA_; CD45RA+CCR7-) memory cells. After four vaccinations, the S-specific CD4+ T cells were mainly T_CM_ and T_EM_ phenotypes, while CD8+ T cells were mostly T_EMRA_ and T_EM_ phenotypes (**Supplementary Figure 5**).

**Figure 3:**
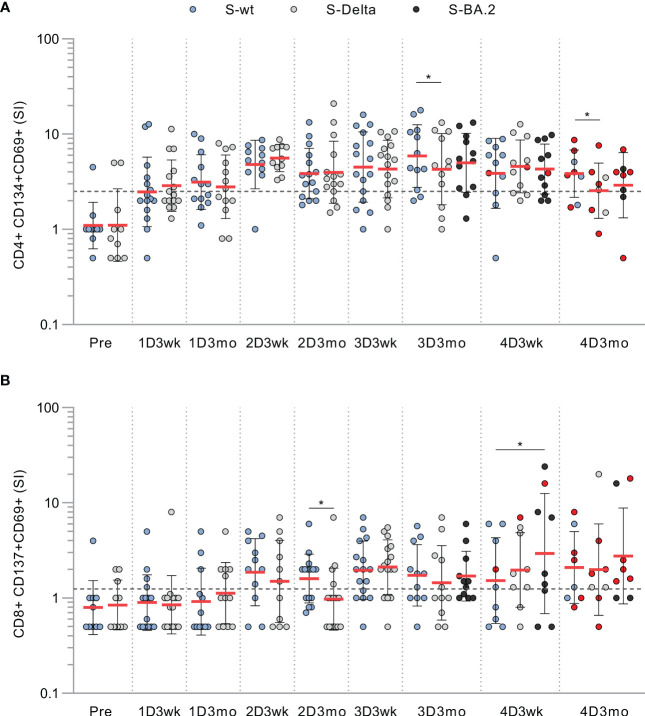
T cell responses to wt and variant peptides in CVID patients. **(A)** CD4+ cell responses against SARS-CoV-2 spike peptide pools from wild-type (S-wt), Delta (S-Delta), and Omicron BA.2 (S-BA.2) variants. **(B)** CD8+ cell responses against SARS-CoV-2 spike peptide pools from wild-type, and Delta and Omicron BA.2 variants. Samples stimulated with all peptide pools are shown and compared with Wilcoxon signed rank test.Red dots represent samples collected after SARS-CoV-2 infection. *p<0.05.

In addition to stimulation with wt spike peptide pool, we stimulated PBMCs collected from CVID patients with Delta and Omicron BA.2 variant spike peptide pools. We found no substantial differences in CD4+ or CD8+ activation against the wt, Delta, or BA.2 variant S peptide pools, although at some time points cross-recognization of Delta was slightly lower than wt (**Figure 3**). Other patient groups had similar results against Delta and Omicron BA.2 spike peptide pools as seen in CVID patients (**Supplementary Figure 6**). We have described in more detail three patients with different outcomes or responses to the vaccination or SARS-CoV-2 infection in the supplementary information. Their individual responses are shown in **Supplementary Figure 7**.

### Secretion of effector cytokines after stimulation with SARS-CoV-2 peptide pools

**Figure 4:**
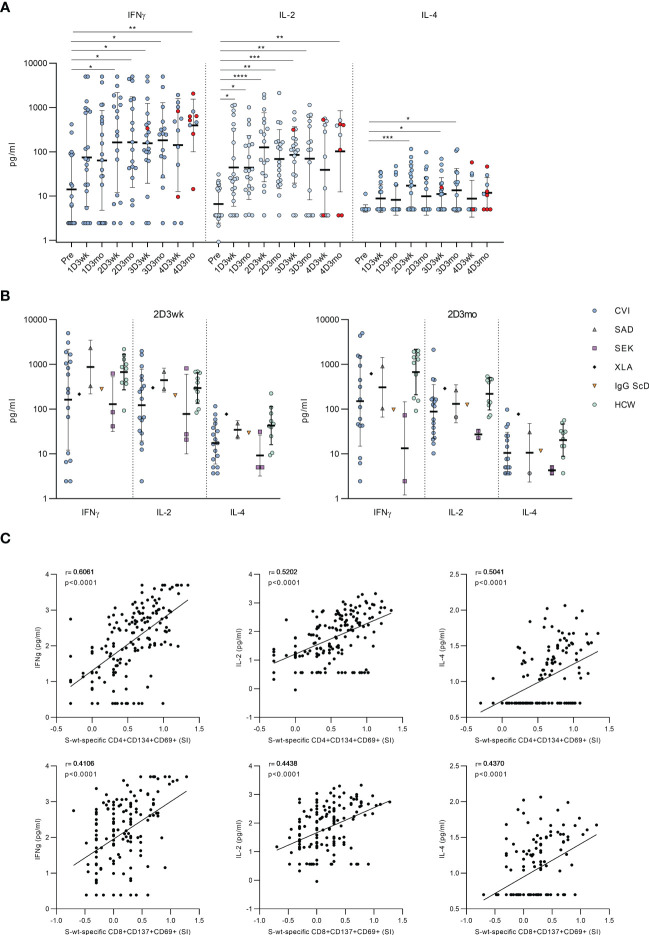
Secretion of cytokines into PBMC culture supernatants after stimulation with SARS-CoV-2 spike-specific peptides. **(A)** Secretion of IFN-γ, IL-2 and IL-4 in PBMCs in CVID patients after stimulation with wt SARS-CoV-2 spike peptide pool. Comparisons between pre-vaccination samples and samples collected after vaccinations were done with Kruskall-Wallis test followed by Dunn’s multiple comparisons test, since some patients lacked individual time points. **(B)** Comparison of production of IFN-γ, IL-2 and IL-4 in all immunodeficiency patients and healthy HCW controls in 2D3wk and 2D3mo samples after stimulation with wt SARS-CoV-2 spike peptide pool. CVID patients were compared to controls with Mann-Whitney U-test. **(C)** Spearman correlation between secreted cytokines (IFN-γ, IL-2 and IL-4) and wt SARS-CoV-2 spike peptide pool -specific CD4+ and CD8+ T cells. *p<0.05; **p<0.01; ***p<0.001; ****p<0.0001.

To further assess the functionality of activated T cells, we measured the levels of secreted cytokines from the supernatants of the stimulated PBMCs. In CVID patients, stimulation with SARS-CoV-2 wt S-peptide pool resulted in a statistically significant increase in the levels of Th1 activation-related cytokines (IFN-γ and IL-2) between the samples taken before the vaccination and after the first or the second vaccination (**Figure 4A**). We also found a statistically significant increase in the secretion of Th2-related cytokine IL-4 between the samples collected before the first vaccination and after the second vaccination, although the IL-4 levels remained lower compared to those of IFN-γ and IL-2 (**Figure 4A**). Cytokine responses in CVID patients resembled those in other immunocompromised patients and immunocompetent HCWs (**Figure 4B**). Notably, IFN-γ levels remained high after each vaccination, while IL-2 and IL-4 levels decreased slightly three weeks after the fourth vaccine dose. Three months after the fourth vaccine dose, all cytokine levels were again elevated, likely due to increased number of infections. S-specific CD4+ T cell response measured with the AIM assay had the strongest correlation with IFN-γ (r=0.6061, p<0.0001), while CD8+ T cell response had the strongest correlation with IL-2 (r=0.4438, p<0.0001) (**Figure 4C**).

The cytokine production in response to SARS-CoV-2 S-peptide pool stimulation was also compared to DMSO-treated PBMCs. The secretion of IFN-γ and IL-2 increased significantly in every post-vaccination sample after S-peptide pool stimulation compared to DMSO-treated PBMCs, while the secretion of IL-4 increased modestly, and the increase was not statistically significant compared to DMSO control (**Supplementary Figure 8**). We also found significant differences in the production of TNF-α and CD8+ T cell activation-related effector molecules, granzyme B and perforin, between DMSO and S-peptide stimulated post-vaccination samples. There was a statistically significant difference between pre- and postvaccination samples in production of Granzyme B, but not in production of TNF-alpha and Perforin (**Supplementary Figure 9**).

## Discussion

Since patients with immunodeficiencies – especially those with CVID – are at an increased risk of getting severe COVID infection (27, 28), it is essential to determine the effectiveness of vaccination against different SARS-CoV-2 variants. In addition, estimating variables of immune protection is important since, at least in the early times of the coronavirus pandemic, commercial immunoglobulin replacement products (IGRTs) did not yet have protective levels of anti-SARS-CoV-2 antibodies (24). Additionally, while many IGRT products had increasing concentration of SARS-CoV-2-specific antibodies with good neutralizing capacity against the ancestral Wuhan lineage, they showed poor neutralization of the Omicron variant (29) Unlike cell-mediated immunity, antibodies are likely not efficiently contributing to virus eradication and protection against severe disease forms. Therefore, the immune protection against severe COVID-19 in CVID patients is most likely dependent on cellular immune responses induced by the vaccination. There have been relatively few studies following long-term immune responses in immunocompromised patients. In immunocompetent individuals, mRNA vaccines have been shown to induce the formation of memory T cells: CD4+ cells mainly of effector memory (T_EM_) and central memory (T_CM_) phenotypes and CD8+ cells of CD45RA+ expressing effector memory (T_EMRA_) phenotype (30, 31). In the present study, we carried out a systematic follow-up of humoral and cellular immune responses after four COVID-19 vaccinations for up to 15 months.

It is of great importance that almost all CVID patients showed increased cell-mediated immune responses against SARS-CoV-2 spike peptides, and the responses are relatively long-lasting and not sensitive to antigenic variation in virus variants. Our data also shows that most patients with hypogammaglobulinemia produce CD4+ and CD8+ cellular responses against all tested viral variant spike peptides already after the first vaccine dose. Furthermore, the responses are strengthened after the second dose and maintained at almost similar levels after additional booster vaccinations over the whole follow-up period. Also, the Th1 type cytokine response (IFN-γ) remained at a steady level after the fourth vaccination. Some previous studies have shown that immunization with a fourth booster dose in immunocompetent individuals and patients with a chronic lymphatic leukemia show increased humoral and cellular immune responses (32, 33). Type 2 and inflammatory cytokine responses were limited based on low IL-4 and TNF-α production in comparison with the DMSO stimulated cells. CD8+ T cell responses were somewhat weaker than CD4+ responses, although some participants showed a good CD8+ T cell activation after the second vaccine dose. This could be due to the suboptimal peptide length (15mers vs. shorter) for antigen presentation for CD8+ T cells as well as the number of circulating cytotoxic CD8+ cells is generally lower than those of antigen activated CD4+ cells (34). CD4+ and CD8+ cellular responses were quite similar between all subgroups of hypogammaglobulinemia, and CVID patients have almost equally strong cell-mediated immune responses compared to immunocompetent HCWs (4). However, some patients with CVID did not show SARS-CoV-2 spike peptide stimulated cellular responses.

In immunocompetent adults, high levels of SARS-CoV-2 S protein-specific antibodies are detected after the second and third vaccinations (25). We also saw S protein-specific IgG antibody responses in many of our CVID patients, especially after the third and fourth vaccine doses. However, increases in antibody levels were rather inconsistent, which may be due to individual differences in patients and the amounts of anti-spike antibodies in commercial immunoglobulin preparations. It is very likely that in most if not in all patients, the detectable anti-spike antibodies are derived from commercial immunoglobulin replacement products. At least XLA patient and rituximab-treated patients cannot produce antibodies, so the measured antibodies must come from external sources.

Our study has some limitations. First, our study group is small – consisting of 23 CVID patients and only one to three patients from other disease entities. However, we have performed a thorough follow-up for each study participant, and we had a good knowledge of the immunological status of each patient before the vaccinations. Second, we used suboptimal-sized peptides to activate the CD8+ cells and thus possibly underestimated the number of CD8+ cells that recognize SARS-CoV-2 peptides. While 15-mers effectively fit into the peptide binding groove of most MHC class II molecules, they are generally too long to fit in MHC class I binding grooves. Shorter peptides like 11-mers and/or 13-mers are more appropriate to detect CD8+ T-cell responses (35). Third, since local municipalities administered the vaccinations in local health care centers, we had no impact of the vaccine type and intervals between the vaccinations. On the other hand, we collected all information related to patient characteristics, types of vaccines, booster intervals, infections and complications and have taken this into consideration in the final analysis.

It was of great importance to observe that despite inadequate COVID-19 vaccine-induced humoral responses, we saw detectable cell-mediated immune responses against the SARS-CoV-2 spike protein that likely provides sufficient protection against a severe form of coronavirus infection in most of the patients. It is also essential to follow the immune status of CVID patients after each vaccine round to determine the need for booster vaccinations.

## Data availability statement

The raw data supporting the conclusions of this article will be made available by the authors, without undue reservation.

## Ethics statement

The studies involving human participants were reviewed and approved by Ethics Committee of the Hospital District of Southwest Finland. The patients/participants provided their written informed consent to participate in this study.Written informed consent was obtained from the individual(s) for the publication of any potentially identifiable images or data included in this article.

## Author contributions

AH, PJ, MM-V, IJ and LK designed the experiments. MM-V and LK recruited the patients and collected the patient information and JL recruited the vaccinated controls. AH, PJ, JH, HJ, SV, and OL performed the experiments. AH and PJ analyzed the data. MM contributed to the data production. AH, PJ, MM-V, IJ and LK wrote the manuscript. All authors contributed to the article and approved the submitted version.
