# Supplementary material for: T cell immunity following COVID-19 vaccination in adult patients with primary antibody deficiency – a 22-month follow-up
Source: Front Immunol. 2023 May 9;14:1146500. doi: 10.3389/fimmu.2023.1146500 (PMC10206403; doi:10.3389/fimmu.2023.1146500)
Supplement: Supplementary file 1 [file DataSheet_1.docx]

**Supplementary Table 1. Combination of COVID-19 vaccines received by immunocompromised patients.**

| **Patient ID** | **DG** | **IGRT** | **Primary vaccination series** | **3rd dose** | **4th dose** |
| --- | --- | --- | --- | --- | --- |
| 1 | CVID | scig | ChAdOx1+mRNA-1273 | mRNA-1273 |  |
| 2 | CVID | scig | BNT162b2+BNT162b2 | BNT162b2 |  |
| 3 | CVID | ivig | ChAdOx1+mRNA-1273 | mRNA-1273 | mRNA-1273 |
| 4 | CVID | scig | BNT162b2+BNT162b2 |  |  |
| 5 | CVID | scig | ChAdOx1+mRNA-1273 | mRNA-1273 | BNT162b2 |
| 6 | CVID | ivig | ChAdOx1+mRNA-1273 | mRNA-1273 |  |
| 7 | CVID | scig | ChAdOx1+BNT162b2 | BNT162b2 | BNT162b2 |
| 8 | CVID | scig | BNT162b2+BNT162b2 | BNT162b2 |  |
| 9 | CVID | ivig | ChAdOx1+mRNA-1273 | BNT162b2 | BNT162b2 |
| 10 | CVID | ivig | ChAdOx1+mRNA-1273 | mRNA-1273 | mRNA-1273 |
| 11 | CVID | scig | BNT162b2+BNT162b2 | mRNA-1273 | BNT162b2 |
| 12 | CVID | scig | ChAdOx1+ChAdOx1 | BNT162b2 | BNT162b2 |
| 13 | CVID | scig | ChAdOx1+BNT162b2 | BNT162b2 |  |
| 14 | CVID | scig | ChAdOx1+ChAdOx1 | mRNA-1273 | BNT162b2 |
| 15 | CVID | scig | ChAdOx1+BNT162b2 | BNT162b2 | mRNA-1273 |
| 16 | CVID | ivig | BNT162b2+BNT162b2 | BNT162b2 | BNT162b2 |
| 17 | CVID | scig | ChAdOx1+mRNA-1273 | mRNA-1273 | mRNA-1273 |
| 18 | CVID | scig | ChAdOx1+ChAdOx1 | BNT162b2 | BNT162b2 |
| 19 | CVID | scig | ChAdOx1+BNT162b2 | BNT162b2 | BNT162b2 |
| 20 | CVID | ivig | BNT162b2+BNT162b2 | BNT162b2 | BNT162b2 |
| 21 | CVID | scig | ChAdOx1+ChAdOx1 | BNT162b2 | BNT162b2 |
| 22 | CVID | ivig | ChAdOx1 |  |  |
| 23 | CVID | scig | BNT162b2+BNT162b2 | BNT162b2 | BNT162b2 |
| 24 | XLA | scig | BNT162b2+BNT162b2 | BNT162b2 | BNT162b2 |
| 25 | SAD | scig | BNT162b2+BNT162b2 | BNT162b2 |  |
| 26 | SAD | scig | ChAdOx1+ChAdOx1 | BNT162b2 | BNT162b2 |
| 27 | SHG | scig | ChAdOx1+BNT162b2 | BNT162b2 |  |
| 28 | SHG | scig | BNT162b2+BNT162b2 | BNT162b2 | BNT162b2 |
| 29 | SHG | ivig | BNT162b2+BNT162b2 | BNT162b2 | BNT162b2 |
| 30 | IgG ScD | ivig | BNT162b2+BNT162b2 | BNT162b2 | BNT162b2 |
| 31 | SAD | ab prof | BNT162b2+BNT162b2 | BNT162b2 | BNT162b2 |

DG; diagnosis, IGRT; Immunoglobulin Replacement Therapy, Ab prof; Antibiotic Prophylaxis

**Supplementary Table 2. Antibodies conjugated with fluorochromes used in activation induced marker (AIM) assay.**

| **Antibody** | **Fluorochrome** | **Manufacturer** | **Cat#** |
| --- | --- | --- | --- |
| Anti-human CD45 | APC-eFluor780 | Invitrogen/Life technologies | 47-0459-42 |
| Anti-human CD3 | eFluor506 | Invitrogen/Life technologies | 69-0038-42 |
| Anti-human CD4 | eFluor450 | Invitrogen/Life technologies | 48-0049-42 |
| Anti-human CD8a | PerCP-eFluor710 | Invitrogen/Life technologies | 46-0087-42 |
| Anti-human CD69 | PE | BD Biosciences | 555531 |
| Anti-human CD134 | PE/Cyanine7 | BioLegend | 350012 |
| Anti-human CD137 | APC | BioLegend | 309810 |
| Anti-human CD45RA | Brilliant Violet 785 | BioLegend | 304140 |
| Anti-human CD197 (CCR7) | PE/Dazzle 594 | BioLegend | 353236 |

**
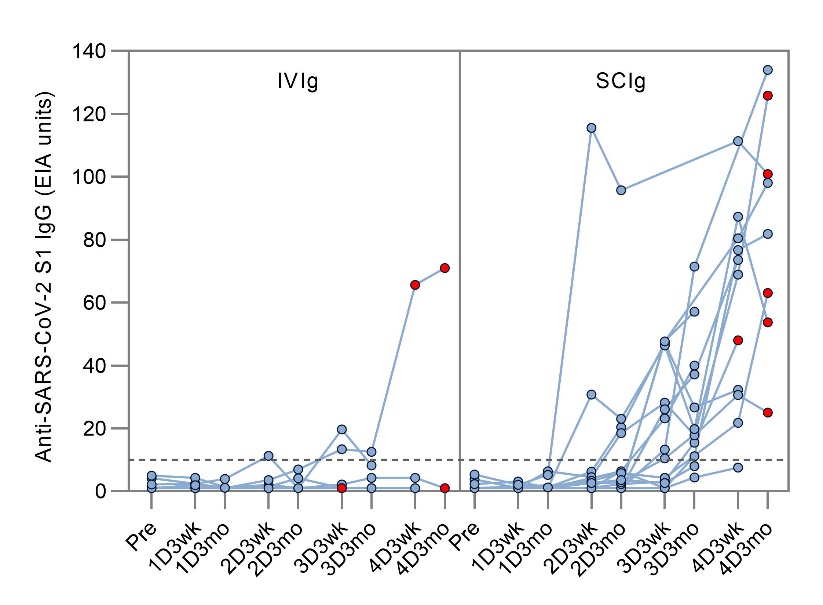
**

**Supplementary Figure 1. Humoral responses in COVID-19 vaccinated CVID patients receiving either intravenous (IV) or subcutaneous (SC) immunoglobulin (Ig) replacement therapy.** Most of the CVID patients that had detectable levels of anti-SARS-CoV-2 S1 antibodies were on subcutaneous immunoglobulin therapy. Red dots represent samples collected after SARS-CoV-2 infection.

**
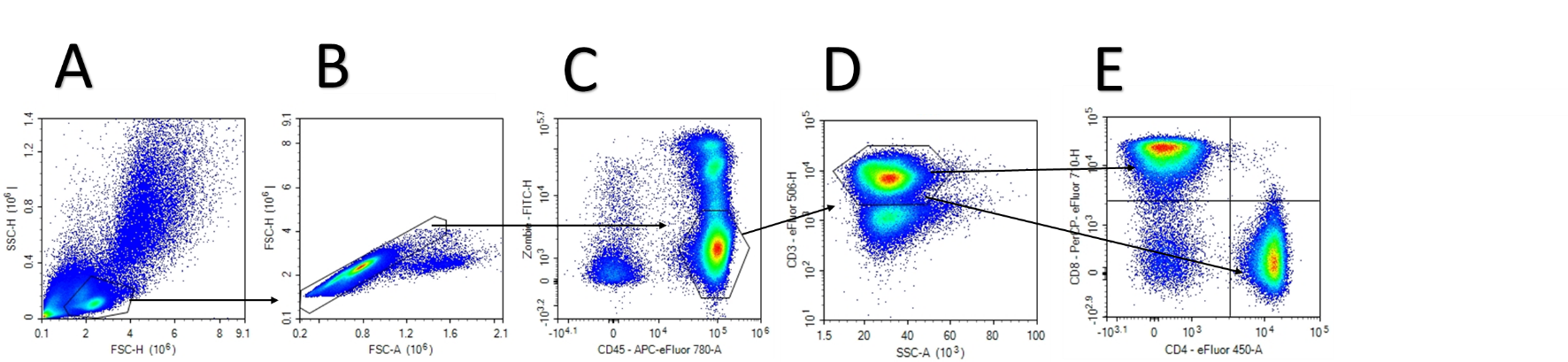
**

E

**Supplementary Figure 2. Gating strategy for selecting CD4+ and CD8+ cells. A.** Selection of lymphocyte population from all PBMCs in forward scatter (FSC) vs side scatter (SSC) plot. **B.** Selection of singular cells in FSC-A vs FSC-H plot. **C.** selection of live CD45+ cells, that are Zombie green dye negative. **D.** Selection of CD3+ T cells. **E.** Selection of CD4+ and CD8+ cells.

**
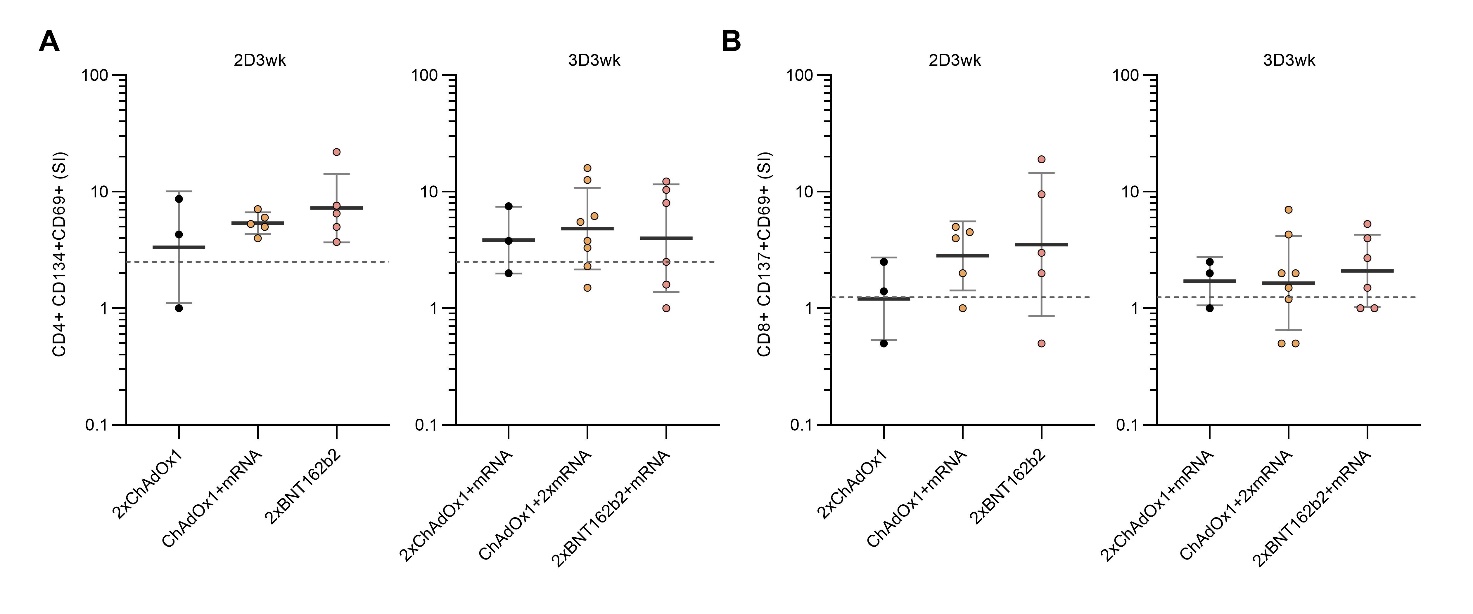
**

**Supplementary Figure 3. T cell responses against SARS-CoV-2 wild type (wt) in CVID patients vaccinated twice with ChAdOx1, ChAdOx1 and mRNA (BNT162b2 or mRNA-1273) vaccine, or BNT162b2. A.** CD4+ responses in three weeks after the second vaccination (2D3wk) and three weeks after the third vaccination (3D3wk). **B.** CD8+ responses in the same time points. The difference between various vaccine combinations was compared with Kruskall-Wallis test followed with Dunn’s multiple comparisons test; however, there is no difference. Data is presented as stimulation indices (SI); number of spike peptide pool activated cells compared to DMSO stimulated control cells.

**
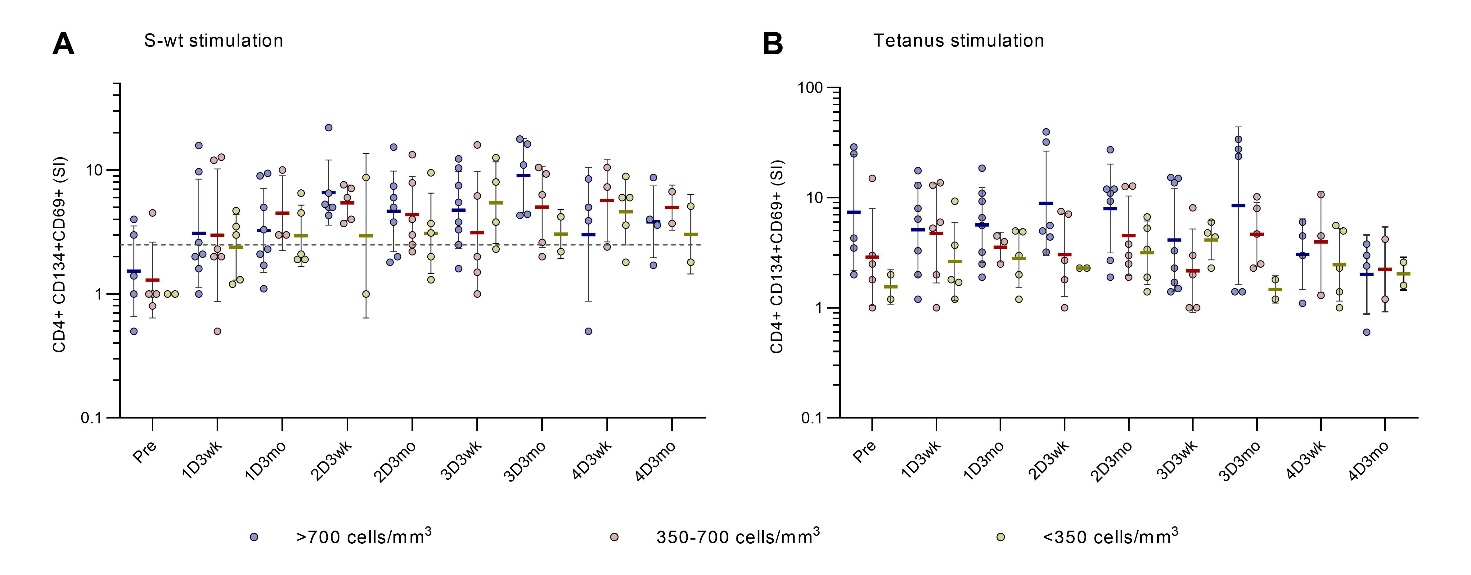
**

**Supplementary Figure 4. CD4+ T cell responses of CVID patients with varying CD4 counts at the time of diagnosis**. **A.** SARS-CoV-2 wt spike and **B.** tetanus toxoid-specific CD4+ T cell responses in patients divided into groups of CD4 count over 700 cells/mm^3^, 350-700 cells/mm^3^ and under 300 cells/mm^3^. Patients with higher CD4+ count have better CD4+ responses against tetanus toxoid and SARS-CoV-2 wt spike peptide pool in most of the time points. Cut-off value for S-specific CD4+ T cells is represented with dotted line.


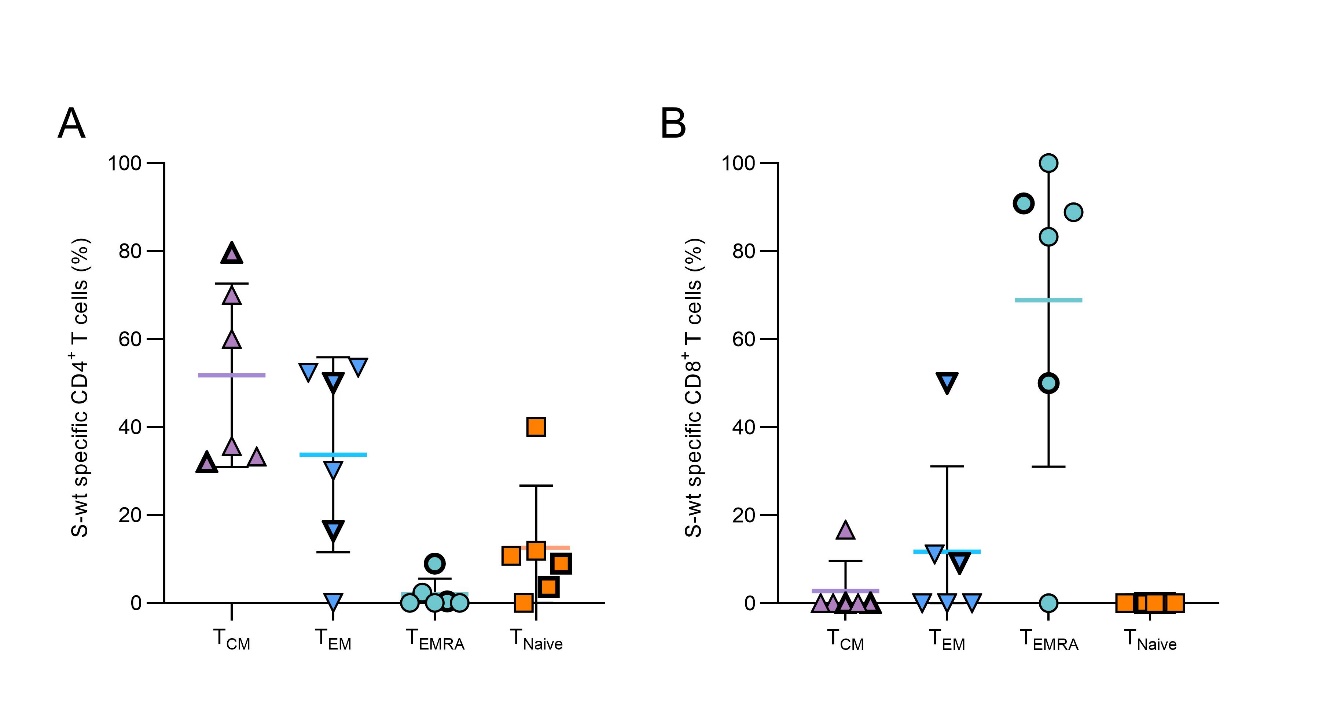


**Supplementary Figure 5.** Distribution of spike-specific **A.** CD4+ and **B.** CD8+ T cells into memory subsets in samples collected from CVID patients after the fourth vaccine dose. Four samples were collected three weeks after the fourth dose and two samples were collected three months after the fourth dose (bolded symbol). T_CM_; central memory, T_EM_; effector memory, T_EMRA_; T effector memory CD45RA+.

**
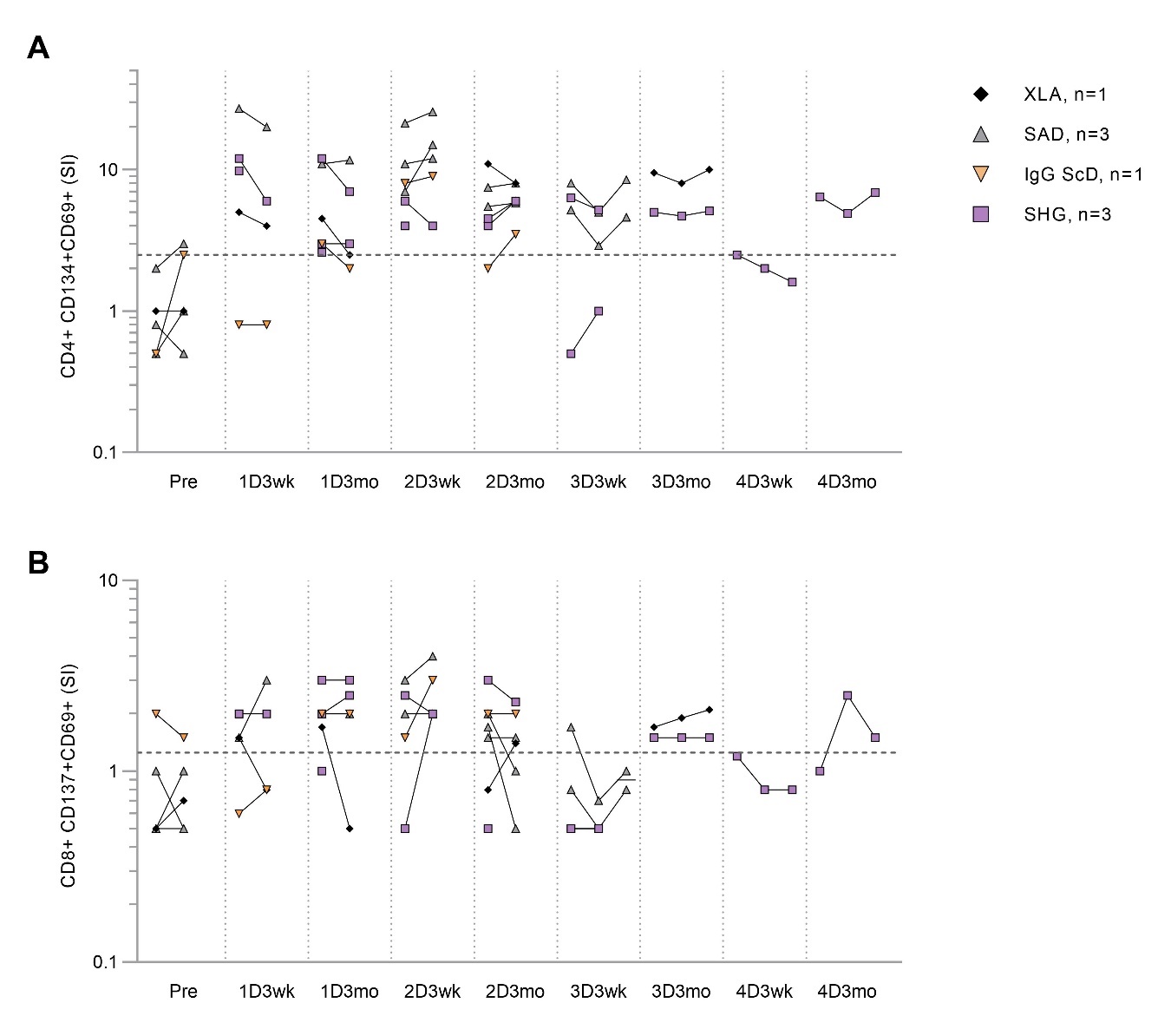
**

**Supplementary Figure 6. T cell responses against SARS-CoV-2 wt and variant peptide pools in patients with different types of hypogammaglobulinemia. A.** CD4+ T cell responses against wt, Delta, and Omicron BA.2 spike peptide pools. **B.** CD8+ T cell responses against wt, Delta, and Omicron BA.2 spike peptide pools. Responses against wt (left), Delta (right, up to 2D3mo, middle) and BA.2 (right 3D3wk to 4D3mo) peptide stimulated in individual samples are connected with lines and are shown in the previously mentioned order. The data is presented as stimulation indices (SI).

**
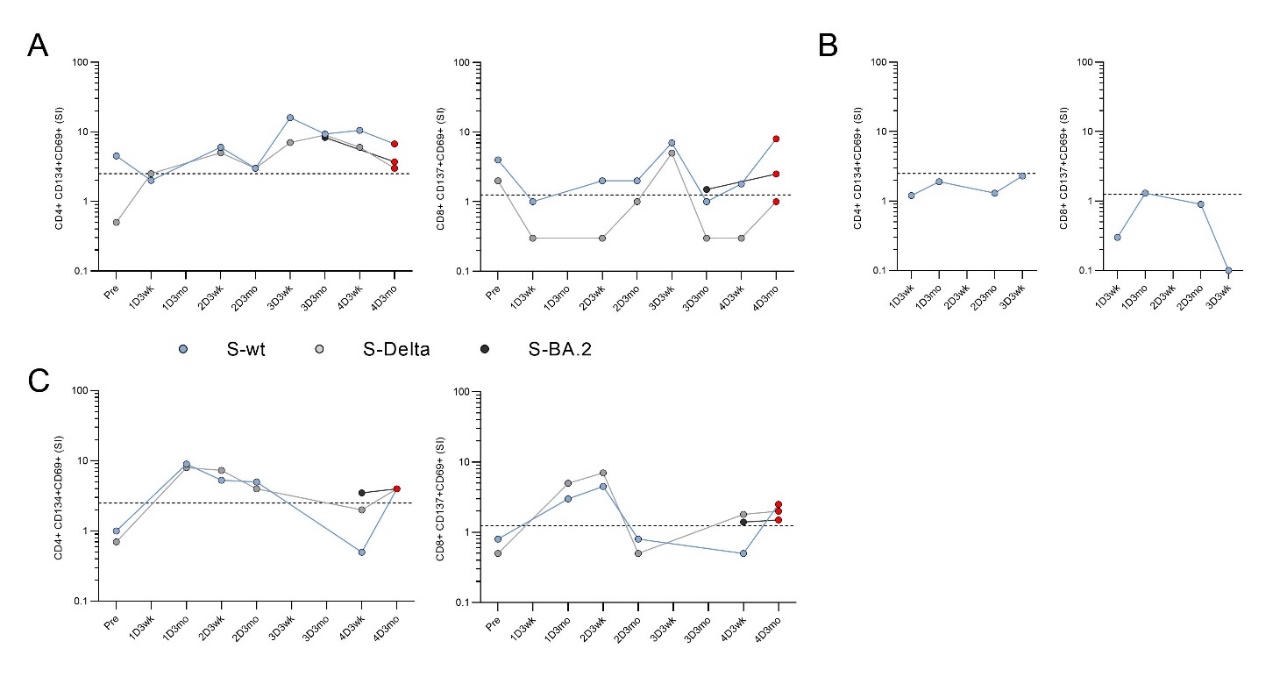
**

**Supplementary Figure 7. SARS-CoV-2 S-specific CD4+ and CD8+ T cell responses of specific COVID-19 vaccinated CVID patients through the study period.** Red dots represent samples collected after SARS-CoV-2 infection.

A. Patient 11: A 53-year-old female patient who was well until the age of 35, after which she started to have recurrent respiratory infections, bronchitis, and sinusitis 6 to 8 times a year. Radiological findings in computed tomography showed bronchiectasis in the lungs. The concentration of IgG, IgA and IgM was low, and she had no serotype-specific response to unconjugated pneumococcal vaccine. However, there was no defect in T cell functions. IVIG was started combined with azithromycin prophylaxis during winter months. She has been relatively healthy after starting immunoglobulin replacement therapy. She received four SARS-CoV-2 vaccinations in February 2021, May 2021, September 2021 and in January 2022. She suffered a mild SARS-CoV-2 infection in February 2022.

B. Patient 10: A 53-year-old male patient, who was diagnosed with CVID at the age of 32. He suffered from ITP and recurrent respiratory infections. Radiological findings showed interstitial lung disease and splenomegaly. Serum IgA, IgG and IgM were below reference values. He had no serotype-specific response to unconjugated pneumococcal vaccine. IVIG was started for CVID and low dose prednisolone because of the pulmonary disease. Respiratory infections were controlled during IVIG therapy, but ITP episodes, however, occurred repeatedly. Rituximab was an effective treatment against ITP episodes for a few years. The latest rituximab dose was administered in August 2021. The patient was working full-time and in general he felt healthy. He had received four SARS-CoV-2 vaccinations in February 2021, May 2021, September 2021, and January 2022. Nevertheless, in April 2022, he got infected with SARS-CoV-2 and developed severe ARDS and finally died of SARS-CoV-2 induced respiratory failure.

C. Patient 14: A 45-year-old female, who was diagnosed with CVID at the age of 40. She suffered from recurrent sinusitis and infections of frontal sinuses and had a functional endoscopic sinus surgery (FESS) operation in 2016. Serum IgA, IgG, and IgM were clearly below reference values, serum IgG was 0,9 g/l at the time of diagnosis. She had no serotype-specific response to unconjugated pneumococcal vaccine. IVIG was started for CVID and after which the upper respiratory infections were controlled. The patient is working full time as a special education teacher and since 2017 IGRT is performed subcutaneously monthly. She is generally healthy and has no other medication. She received four SARS-CoV-2 vaccinations in March 2021, in May 2021, in September 2021 and January 2022. Nevertheless, in February 2022, she got infected with SARS-CoV-2, but with relatively mild symptoms.

D

C


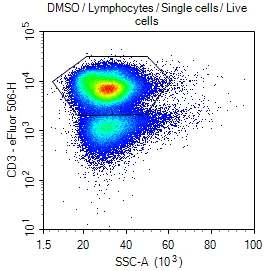

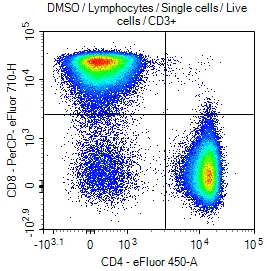


*
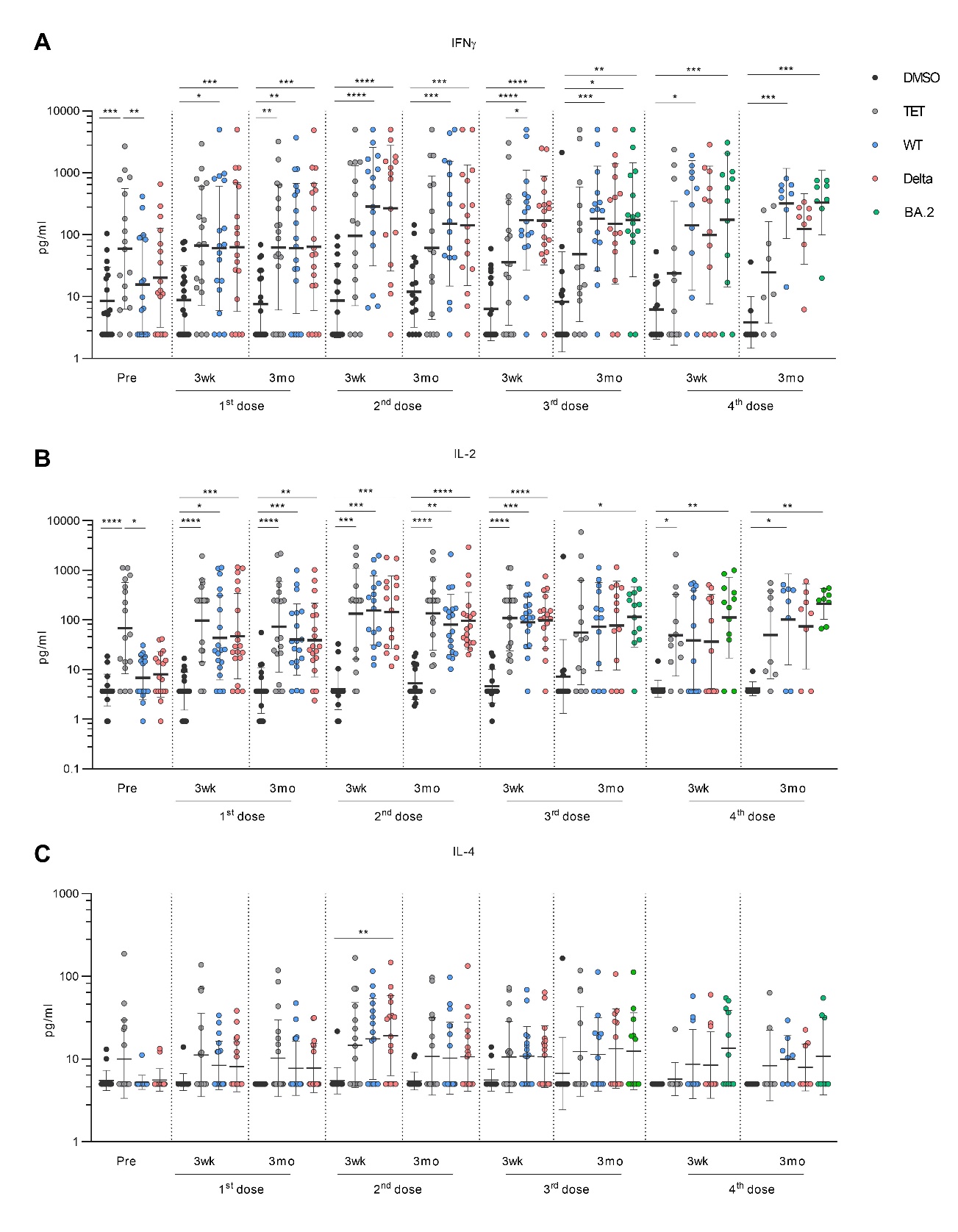
***Supplementary Figure 8. Secretion of cytokines after stimulation of PBMCs with DMSO, tetanus toxoid (TET) and SARS-CoV-2 wild type (WT), Delta, or Omicron BA.2 spike peptide pools in COVID-19 vaccinated CVID patients. A.** Secretion of IFNγ **B.** Secretion of IL-2 **C.** Secretion of IL-4. Statistical significances were compared with the Kruskal-Wallis test followed by Dunn’s multiple comparisons test since some participants were missing samples from individual time points. *p<0.05; **p <0.01; ***p <0.001; ****p <0.0001.

*
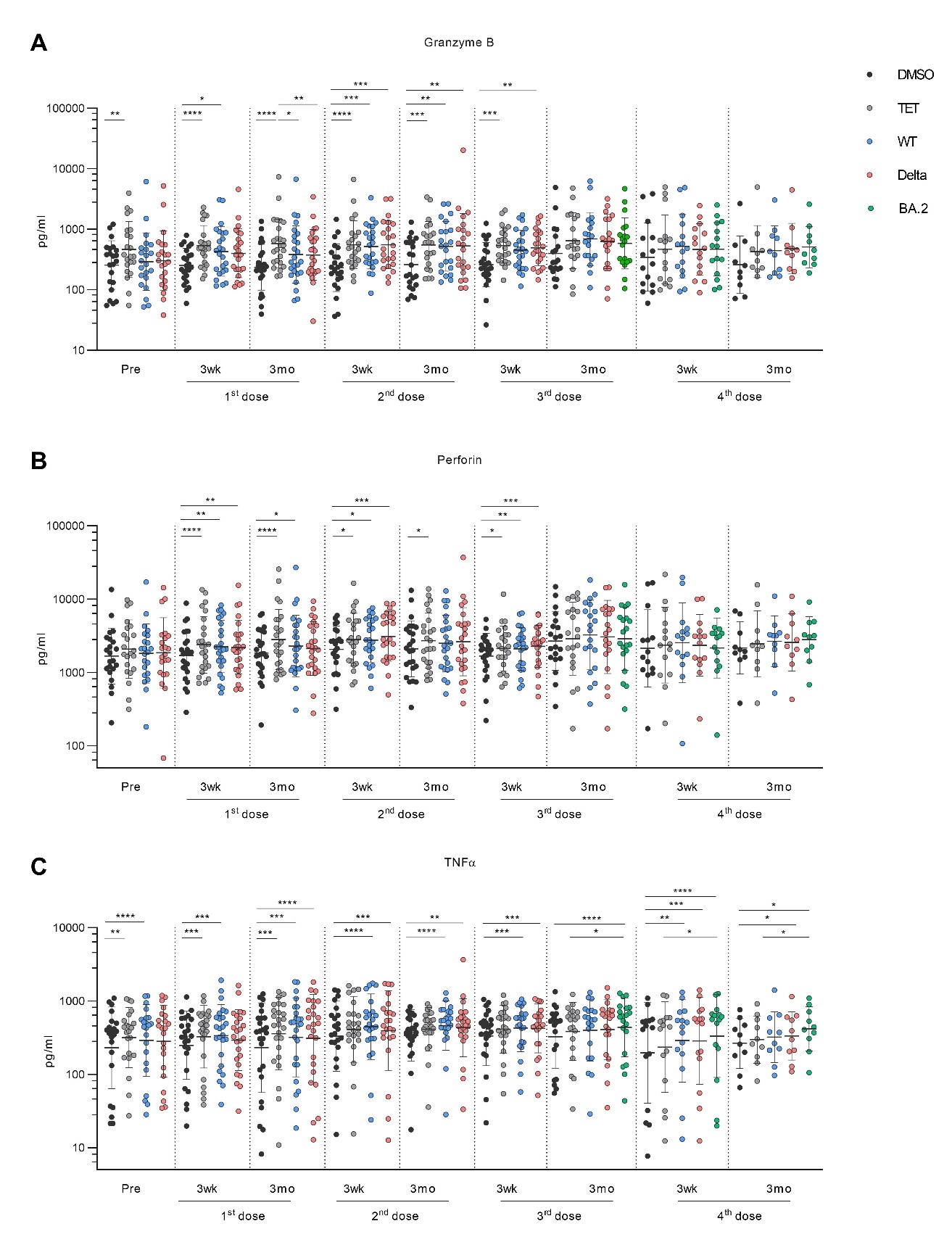
*

**Supplementary Figure 9. Secretion of cytokines and effector molecules after stimulation of PBMCs with DMSO, tetanus toxoid (TET) and SARS-CoV-2 wild type (WT), Delta, or Omicron BA.2 spike peptide pools in COVID-19 vaccinated CVID patients. A.** Secretion of Granzyme **B.** Secretion of Perforin. **C.** Secretion of TNFα. Statistical significances were compared with the Kruskal-Wallis test followed by Dunn’s multiple comparisons test since some participants were missing samples from individual time points. *p<0.05; **p <0.01; ***p <0.001; ****p <0.0001.
